# (Un)Tying the Knot: Oxidative Stress, Inflammatory Markers, and Lipid Status in Dogs with Hypercortisolism

**DOI:** 10.3390/ani14233476

**Published:** 2024-12-02

**Authors:** Lazar Karić, Filip Janjić, Kristina Spariosu, Darko Davitkov, Vanja Krstić, Milica Kovačević Filipović, Milena Radaković

**Affiliations:** 1 Department of Equine, Small Animal, Poultry and Wild Animal Diseases, Faculty of Veterinary Medicine, University of Belgrade, Bulevar Oslobodjenja 18, 11000 Belgrade, Serbia; lazar.karic@vet.bg.ac.rs (L.K.); davitkov@vet.bg.ac.rs (D.D.); vanjak@vet.bg.ac.rs (V.K.); 2 Department for Immunochemistry and Glycobiology, Institute for the Application of Nuclear Energy (INEP), University of Belgrade, Banatska 31b, 11000 Belgrade, Serbia; filip.janjic@inep.co.rs; 3 Department of Pathophysiology, Faculty of Veterinary Medicine, University of Belgrade, Bulevar Oslobođenja 18, 11000 Belgrade, Serbia; kristina@vet.bg.ac.rs (K.S.); milica@vet.bg.ac.rs (M.K.F.)

**Keywords:** hyperadrenocorticism, oxidative damage, uric acid, systemic immune-inflammatory markers lipid status, dogs

## Abstract

The aim of this study was to revisit the complex relationship between inflammation, oxidative stress and lipid metabolism in dogs with hypercortisolism (HC). Fourteen dogs newly diagnosed with HC and an equal number of healthy counterparts of similar age and markers of oxidative stress (AOPP, TBARS, TAC, GSH, PON-1, and UA) and inflammation (NLR, PLR, SII, HPT, CHE, CP, and Hcy) were included in the study. To determine the lipid profiles, cholesterol, and triglyceride levels were measured, and the electrophoretic separation of lipoproteins was performed. The results revealed that dogs with HC had higher levels of AOPP and TBARS, but only greater levels of GSH among antioxidants. Uric acid levels were higher in HC dogs, suggesting a pro-oxidative role. Elevated NLR, PLR, SII, and HPT levels were detected, but they did not seem to be associated with inflammation. Notable changes were detected in the HDL fraction, alongside hypercholesterolaemia and hypertriglyceridaemia. Correlation analysis revealed links between lipid markers and both oxidative stress and inflammatory indices. In conclusion, the data acquired may prove useful in further understanding of the intricate pathophysiology of Cushing’s syndrome.

## Simple Summary

Hypercortisolism (HC) is a common endocrine disorder in dogs characterised by long-lasting high levels of cortisol, which is the most important hormone in stress response. Thus, in both animals and humans, HC is a good model for studying the detrimental effects of chronic exposure to stress. However, up to the present time, the full range of mechanisms by which it exerts its negative effects remains unexplained. In this study, our goal was to detect and describe the biochemical alterations caused by cortisol, and how these may compound and create a destructive cycle. Some of these changes can be detected early in the course of the disease and therefore aid in diagnosis.

## 1. Introduction

Hypercortisolism (HC) is one of the most frequent endocrine disorders in dogs [1]. Chronic excessive circulating cortisol levels lead to clinical signs such as polydipsia, polyuria, polyphagia, abdominal distension, and alopecia [2]. Untreated HC may result in serious complications, including increased susceptibility to infections, impaired wound healing, as well as potentially fatal pulmonary thromboembolism [1]. These signs may affect both the dogs and their owners’ daily routines. The unifying pathological event is increased metabolic rate, specifically catabolism, insulin resistance, dyslipidaemia, and endothelial dysfunction. The potential contradictory effects of hormones, oxidative stress, and inflammation in endocrine disorders contribute to the complexity of HC.

Oxidative stress, defined as an imbalance between the production of reactive oxygen species (ROS) and the body’s antioxidant defence, has been implicated in various human endocrine disorders, including Cushing’s syndrome [3]. In canine HC, studies examined only the markers of oxidative damage, resulting in inconsistent findings [4,5].

The role of inflammation in canine HC also remains a subject of debate. So far, acute phase proteins (APPs) were used to assess inflammation in HC dogs, but the results were inconclusive. Some researchers argue that increased glucocorticoid (GC) levels blunt the acute phase response in dogs with HC and concurrent inflammation [6], while others suggest that higher haptoglobin (HPT) levels are linked to inflammation itself, rather than are directly triggered by GC production [7]. This divergence prompts the need for further investigation into other inflammatory response-related scoring indices to better understand the role of inflammation in HC pathology.

Dyslipidaemia, one of the hallmarks of HC, is marked by altered levels of lipids in the blood, mainly hypertriglyceridaemia and hypercholesterolaemia [8]. It is known that increased oxidative stress and inflammation are linked with impaired lipid metabolism [9,10], but this is unexplored in canine HC.

Overall, understanding the interplay between oxidative stress, inflammation, and lipid status in canine HC patients may provide valuable insights into the underlying pathophysiology of this condition and guide the development of targeted therapeutic interventions. For that purpose, this research aims to (1) assess the oxidative stress and inflammatory markers as well as lipid status in dogs newly diagnosed with HC and healthy controls; and (2) uncover the link between these factors.

## 2. Materials and Methods

### 2.1. Animals

The dogs newly diagnosed with HC presented to the Small Animal Teaching Hospital (Faculty of Veterinary Medicine, University of Belgrade) were enrolled in the study. All owners signed a consent form for the use of the data obtained on their animals for clinical research. Hypercortisolism was made a differential diagnosis in the dogs exhibiting typical clinical signs (polydipsia, polyuria, panting, and polyphagia) and based on physical examination (alopecia, thin skin, pendulous abdomen, and calcinosis). Afterwards, their blood samples were analysed in the hospital laboratory. A confirmatory endocrinological test was carried out if the signalment, clinical signs reported by the owner, physical examination findings, and clinicopathologic data pointed towards HC. The diagnosis was confirmed using either the modified urinary corticoid-to-creatinine ratio with oral dexamethasone suppression [11] or by a combination of urinary corticoid-to-creatinine ratio and the ACTH stimulation test. All the dogs underwent an abdominal ultrasound examination (Vetus 8, Mindray, Shenzhen, China) to further differentiate between pituitary-dependent (PDH), adrenal-dependent (ADH), and iatrogenic HC [2]. In dogs diagnosed with PDH, the mean dorsoventral diameter of the left adrenal gland was 9.16 mm, while of the right one was 8.84 mm. In contrast, in two dogs with iatrogenic HC the adrenal glands were smaller, with a mean dorsoventral diameter of 4.5 mm for the left and 4.25 mm for the right gland. Additionally, on ultrasound evaluation, one dog was found to have a mass affecting the left adrenal gland, characterised by an asymmetrical enlargement of the cranial pole and a mixed echogenicity pattern. The contralateral adrenal measured 4.2 mm.

In this study, 14 dogs with confirmed HC were included: 11 with pituitary-dependent and 2 with iatrogenic HC, and 1 diagnosed with a functional adrenal tumour. The exclusion criteria were (1) previous treatment for HC, and (2) other obvious comorbidities that could not be attributed to HC. The control group consisted of 14 clinically healthy dogs of various breeds, with CBC and biochemistry results within reference intervals.

### 2.2. Sample Collection and Analysis

Whole blood was collected from the dogs via cephalic venipuncture into both EDTA-containing and plain tubes. The samples for CBC were analysed immediately after sampling, on the Mindray BC-5000 Vet Haematology Analyser (Mindray, Shenzhen, China). The neutrophil-to-lymphocyte ratio (NLR) and platelet-to-lymphocyte ratio (PLR) ratio were determined by dividing the absolute neutrophil and platelet counts, respectively, with the absolute lymphocyte count. The systemic immune–inflammatory index (SII) was calculated according to the following formula: platelet × neutrophil/lymphocyte counts. The sera for biochemistry analyses were separated at 4000 rpm for 5 min and analysed within one hour, using commercial kits on an automatic analyser (Mindray BS-240, Shenzhen, China). The biochemical profile included calcium, phosphorus, glucose, alanine aminotransferase (ALT), aspartate aminotransferase (AST), alkaline phosphatase (ALP), gamma–glutamyl transferase (γ–GT), total proteins, albumin, triglycerides, total cholesterol, urea, creatinine, and creatine kinase (CK). Subsequent analyses of cholinesterase (CHE), beta-hydroxybutyrate (BHB), and uric acid (UA) were performed on the same analyser. Homocysteine (Hcy) levels were measured with the commercial chemiluminescent microparticle immunoassay (CMIA) on the ARCHITECT^®^ ci8200 (Abbott, Chicago, IL, USA). The remaining sera were transferred to microtubes and stored at −20 °C for further analyses.

### 2.3. Oxidative Stress Biomarkers

A lipid peroxidation marker, thiobarbituric acid-reactive substances (TBARs), was measured at 535 nm using the method developed by Asakawa and Matsushita [12]. Advanced oxidation protein products (AOPPs) levels were quantified following the protocol by Witko-Sarsat et al. [13], utilising a combination of acetic acid and potassium iodide. Total antioxidant capacity (TAC) in serum was measured using the 2,2′-azino-di-3-ethylbenzthiazoline sulfonate (ABTS)^+^ following the Erel method [14]. Serum reduced glutathione (GSH) concentration was determined according to the modified Ellman’s method [15] with 5,5′-dithiobis-(2-nitrobenzoic) acid (DTNB). The activity of paraoxonase-1 (PON-1) in serum was estimated by utilising 4-nitrophenyl acetate as a substrate [16].

### 2.4. Inflammatory Indices

The HPT level in serum was assessed following a modified protocol by Jones et al. [17] which determines the peroxidase activity of the haptoglobin–haemoglobin complex. The level of ceruloplasmin (CP) in the serum was assessed through its p-phenylenediamine (PPD) oxidase function, as described by Hussein et al. [18], and the absorbance of the resulting purple product was recorded at 530 nm.

### 2.5. Substrate Zymography

Gelatinolytic matrix metaloproteinase-2 and 9 (MMP-2 and MMP-9) were detected by sodium dodecyl sulphate–polyacrylamide gel electrophoresis (SDS-PAGE) [19]. Five-fold diluted sera were mixed with the sampling buffer (125 mM Tris, 4% SDS, 20% glycerol, 0.02% bromophenol blue, pH 6.8) at equal volumes, and 10 µL of each sample was loaded on 8% polyacrylamide gel, with the addition of 2% gelatine. Foetal calf serum (10%) (FCS, Gibco, Thermo Fisher Scientific, Waltham, MA, USA) was used as a loading control. Separation was performed for 15 min at 80 V and 1 h at 120 V. After washing in 2% Triton X-100 (Fisher Scientific, Pittsburgh, PA, USA), gels were soaked overnight in the developing buffer, at 37 °C. The next day, gels were stained with 0.25% Coomassie brilliant blue G250 (Serva, Heidelberg, Germany), and washed in a destaining solution, until clear bands appeared. ChemiDoc™ Imaging System (2.2.0.08) (Bio-Rad, Hercules, CA, USA) was used for scanning, TotalLab TL120 ^®^ software (1D v2009) for MMP signal evaluation. Levels of MMP-2 and MMP-9 were calculated based on the band measurement, normed by the FCS unit value, and expressed in arbitrary units (AU).

### 2.6. Serum Lipoprotein Agarose Electrophoresis

Agarose strips were prepared as described by Milanović et al. [20]. Depending on their migration distance, canine lipoproteins separate into distinct bands on the gel: chylomicrons, α1- (high density lipoproteins, HDL-2,3), α2- (HDL-1), pre-β (very low-density lipoproteins, VLDL), and β (low density lipoproteins, LDL). Due to the challenge of distinguishing pre-β and β bands in dogs, they were merged and documented as triglyceride-rich lipoproteins (TRLs) [21]. The results were displayed as the relative concentration of lipoproteins found in each band, quantified by ImageJ software (Windows 64-bit Java 8 version; available online: https://imagej.net/ij/download.html (accessed on 23 June 2024)).

### 2.7. Statistical Analyses

The Shapiro–Wilk test was used to check the normality of the data sets. Non-parametric tests were utilised due to the non-normal distributions of the data. The difference in gender distribution between the HC and the control group of dogs was examined by the Chi-squared test. The differences in numerical data between groups were examined by the Mann–Whitney U test. The relationship between oxidative stress, inflammation, and lipid markers in HC dogs was examined by Spearman’s correlation analysis. The statistical analyses were performed using IBM SPSS Statistics 26 software. GraphPad Prism, version 9 (GraphPad, San Diego, CA, USA) was used for the presentation of the Figures.

## 3. Results

### 3.1. Baseline Characteristics

The demographic and clinical data about the dogs included in the study are summarised in Table 1. No significant differences in sex distribution and the average age were found between HC dogs and the control.

Haematology revealed that in the HC group, neutrophil and platelet counts were higher, while the numbers of lymphocytes and eosinophils were lower in comparison with the control. In addition, HC dogs had markedly higher NLR, PLR, and SII values in comparison with healthy subjects (Table 2). The other haematological parameters did not differ between the two groups.

The serum activities of ALP, AST, ALT, γ-GT, and CK were significantly higher in comparison with healthy dogs. Also, in the HC group, the serum concentrations of albumin, phosphorus, and glucose were higher, while the creatinine level was lower compared to the controls. The serum concentrations of total proteins, urea, and BHB did not differ significantly between the groups (Table 3).

### 3.2. Oxidative Stress, Inflammatory Markers and Lipid Status

With regard to the parameters of oxidative damage, in HC dogs AOPP and TBARS levels were higher than in the control. Moreover, higher levels of GSH and UA were recorded in the same group. There were no differences in other antioxidants between the two groups of dogs (Table 4).

**Figure 1:**
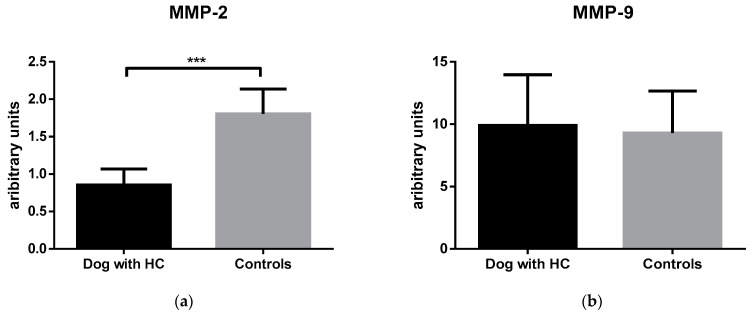
MMP-2 (**a**) and MMP-9 (**b**) levels in dogs with hypercortisolism (HC) and healthy controls. Boxplots represent the interquartile range (box), the median value with (line inside the box), and the data range (horizontal lines extending from the box). *** indicates *p* ˂ 0.001.

Inflammatory indices showed that only HPT was higher in the HC group, while CP, CHE, and Hcy did not differ significantly between the groups. Regarding MMPs activity, MMP-2 was markedly lower in HC dogs (Figure 1). By contrast, MMP-9 did not reveal distinctions between the two groups.

The lipid profile analysis showed elevated levels of cholesterol and triglycerides in the HC group. The percentage of chylomicrons, as well as the TRL, did not differ significantly between the two groups. However, the percentage of HDL-1 was significantly higher in the HC dogs, unlike HDL-2,3, which was lower (Table 5).

### 3.3. Correlation Analysis

**Figure 2:**
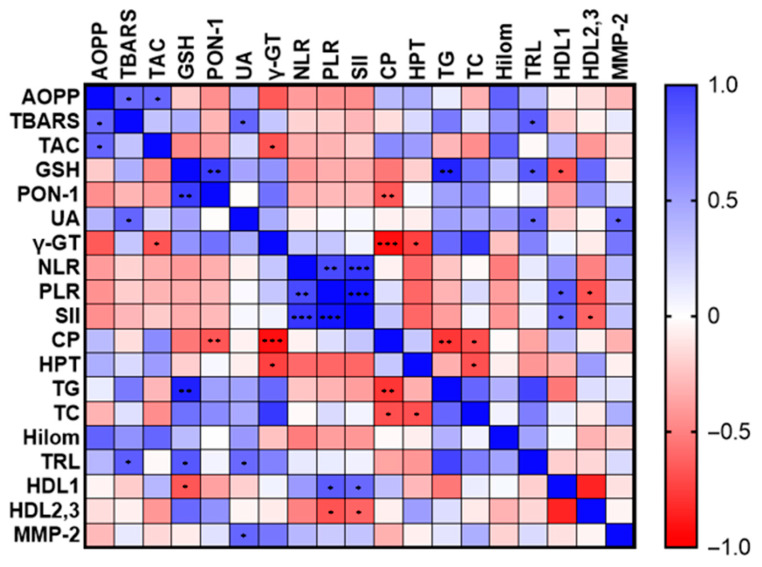
Relationship between oxidative stress, inflammation and lipid markers in dogs with hypercortisolism (HC). The colour gradient reflects the value of the correlation coefficient. * indicates *p* ˂ 0.05; ** indicates *p* ˂ 0.01; and *** indicates *p* ˂ 0.001.

Correlations between oxidative stress, inflammation and lipid markers in dogs with HC are presented in Figure 2. TBARS concentrations showed a positive correlation with AOPP (r = 0.604, *p* = 0.029), UA (r = 0.621, *p* = 0.024), and TRL (r = 0.654, *p* = 0.015). TAC levels were positively correlated with AOPP (r = 0.612, *p* = 0.020), but negatively with γ-GT (r = −0.667, *p* = 0.049). UA concentration was associated with MMP-2 (r = 0.616, *p* = 0.025), and TRL (r = 0.604, *p* = 0.022). The negative correlations of HPT with TC (r = −0.685, *p* = 0.029), and γ-GT (r = −0.738, *p* = 0.037) were also observed. The NLR showed a strong positive correlation with PLR (r = 0.734, *p* = 0.007) and MMP-9 (r = 0.685, *p* = 0.014). PLR positively correlated with HDL-1 (r = 0.657, *p* = 0.020) and negatively with HDL-2,3 (r = −0.671, *p* = 0.020). Similarly, the SII positively correlated with MMP-9 (r = 0.741, *p* = 0.006), and HDL-1 (r = 0.601, *p* = 0.043), but negatively with HDL-2,3 (r = −0.608, *p* = 0.040).

## 4. Discussion

This research focused on the oxidative stress and antioxidant defence levels, inflammatory markers, and lipid status in dogs newly diagnosed with HC, and their potential correlation. Several revelations have been made: (1) dogs with HC had higher levels of AOPP and TBARS, along with increased antioxidant GSH in comparison with the healthy control; (2) in canine HC higher levels of UA exerted pro-oxidant activity; and (3) lipid markers had a more significant correlation with oxidative stress and inflammatory markers than was the correlation between the latter two.

We observed that newly diagnosed HC dogs had higher levels of AOPP compared to their healthy counterparts. According to the veterinary literature, AOPP as a marker of protein oxidation was not assessed in dogs with HC. However, Kim et al. [20] failed to detect higher protein oxidation quantified by carbonylated proteins level in dogs with HC. This could be attributed to the fact that those dogs had been undergoing trilostane therapy for two months, which potentially influenced the findings. In vitro and in vivo studies indicate that GC can cause oxidative stress when released in increased amounts and chronically/for a long time [22,23,24,25]. Various mechanisms for GC-induced oxidative stress are proposed such as uncoupling, proton leak, mitochondrial dysfunction and increase catabolic activity [25,26,27]. We supposed that in the oxidative stress milieu, myeloperoxidase (MPO) from higher number of neutrophils is released, and forms chlorinated oxidants, which together with plasma proteins give rise to AOPP [28]. AOPPs may also activate nicotinamide adenine dinucleotide phosphate (NADPH) oxidase, promoting ROS and endothelial dysfunction [29].

Another piece of evidence of oxidative damage in HC dogs was higher TBARS, marker of lipid peroxidation (LP), which is in line with the study by Soares et al. [4]. It was found that medical treatment could decrease LP by stabilising HC, thus underscoring the role of LP in disease progression. One of the clinical manifestations of canine HC are dermal alterations such as alopecia, thinning of the skin, and comedones, which were present in more than one-third of our dogs with HC. MDA in the serum and tissue from *alopecia areata* scalp biopsies were higher in HC patients than in healthy subjects [30,31]. Also, higher levels of LP were noted in comedone samples [32], implying that skin alterations in HC patients may be additional foci of increased oxidative stress. Certain underlying conditions, e.g., hyperglycaemia, may also contribute to the development of oxidative stress in HC-affected dogs [33].

Regarding antioxidant status, TAC levels and PON-1 activity remained unchanged in newly diagnosed HC dogs in comparison to healthy ones. This mirrors findings by Karamousis et al. [34], who reported increased oxidative stress measured by 15-F2t-Isoprostane without changes in TAC in patients with Cushing’s syndrome. Nevertheless, we found that GSH levels were elevated in dogs with HC. Similarly, in iatrogenic HC, GSH was upregulated in mesenchymal stem cells [35]. Despite the insignificant difference in other antioxidant parameters (TAC and PON-1) between the groups, a positive correlation between AOPP and TAC, as well PON-1 and GSH were noticed. This implies that antioxidant capacity observed at the time of HC diagnosis was triggered/stimulated, but insufficient to combat the oxidative damage.

One of the interesting findings in this study was a considerably higher uric acid (UA) concentration in the HC group compared with the control. UA is the final byproduct of the catabolism of purine, which primarily source from animal proteins. Higher UA levels could be explained by increased proteolysis triggered by GCs. So far, serum UA concentrations in dogs with HC were not described. Given that UA exerts antioxidant and pro-oxidant properties [36], we set out to explore how its increased levels are linked to other biochemical markers. There is evidence that UA stimulates ROS production thought activation of NADPH oxidase and an increased lipid oxidation [36], which was highlighted here by the positive correlation between UA and TBARS. Additionally, increased UA may promote MPO release from neutrophils, leading to UA oxidation [37], and the generation of reactive intermediates, thereby fostering oxidative stress. Hence, it appears that UA in dogs with HC display a pro-oxidative behaviour.

Despite extensive data in human medicine, veterinary research lacks information on systemic immune–inflammatory markers (NLR, PLR, and SII), especially on their correlation with oxidative stress and lipid markers. These markers result from simultaneous changes in blood cell counts, which are influenced by various immunologic, neuroendocrine, humoral, and biological factors [38]. Due to the dynamic nature of the immune response in stress, the interpretation of the haematological ratio results requires caution. Clinical changes in dogs with HC take time to develop, while the changes in the above-mentioned markers seem to respond more rapidly to GC level. It has been proven that physical exercise, being a type of physiological stress, can promptly raise the values of NLR, as well as PLR and SII in people [39]. These findings may be used for early screening of chronic stress exposure in dogs, but further studies are required to establish the cut-off values that distinguish physiological from pathological stress.

Our findings revealed markedly higher levels of NLR and PLR in HC dogs compared to the control, which agrees with previous findings [40]. Higher NLR in HC group is probably the result of stress leucogram (neutrophilia and lymphopaenia) induced by higher GCs level. The association between NLR and cortisol levels has been proven [41]. Higher PLR may stem from thrombocytosis in the HC group, possibly triggered by GC-induced activation of the thrombopoietin receptor or decreased platelet removal from circulation due to inhibited mononuclear phagocyte function [42]. SII is a newly identified marker for systemic inflammation calculated from neutrophils, lymphocytes and platelet counts. In canine HC, in the current research SII was found to be higher than in the control, which is comparable to higher levels of SII found in humans with Cushing’s syndrome [43]. Correlation analysis suggested that there was an association between PLR and SII with HDL levels in dogs with HC, highlighting the role of HDL in modulating platelet function [44]. Patients with HC were discovered to have activated platelets [34], rendering them prone to pro-thrombotic and pro-inflammatory states.

Regarding the APPs assessed here, only HPT was higher in HC dogs in comparison to healthy controls, which is consistent with the results of previous research [5,45]. Even though there was an increase in HPT levels, the value does not exceed the reference range (>3 g/L), suggesting that, given our previous results, the rise in this APP did not result from inflammation in HC. We supposed that increased GCs levels in HC could trigger higher HPT concentrations through the IL-6 as a major mediator for HPT gene expression [46,47]. Interestingly, the concentrations of Hcy, which could be used as a negative acute-phase reactant [48], did not differ between the groups, pointing that HC dogs without comorbidities, despite oxidative damage, did not develop an inflammatory component of the disease.

Matrix metalloproteinases (MMPs) are endopeptidases with a great potential for modifying the inflammatory response [49]. Our assessment focused on the levels of MMP-2 and MMP-9, known as gelatinases, which are most effective in breaking down gelatine and laminin, and are crucial in the process of tissue injury and healing. We detected lower levels of MMP-2 in HC dogs in comparison with the control. Corroborating our data, MMP-2 was shown to be suppressed in the visceral adipose tissue of dogs receiving ACTH [50]. The lower levels of MMP-2 in HC dogs may be explained by the fact that HPT reduced the activities of gelatinases and the migration of fibroblasts, crucial for tissue regeneration [51]. Impaired wound healing in HC patients further supports this theory [35]. Also, lower MMP-2 levels in HC dogs could have resulted from its impact on vascular bed remodelling, given that endothelial cells are known to release MMP-2 [52].

Correlation analysis failed to detect a relationship between oxidative stress and inflammatory markers, which suggested that in HC these two mechanisms are not connected. This aligns with our findings, which revealed a lack of significant inflammatory responses in HC dogs. Discussing similarities between people and dogs, it can be underlined that people with HC also experience oxidative–antioxidant status imbalance [34,53,54]. Similarly, the inflammation regarding acute phase response does not appear to be neither in dogs nor in humans. CRP levels in HC remain consistent in both dogs and humans. However, in canine HC, elevated HPT levels have been observed, potentially reflecting its antioxidant properties. This APP, however, has yet to be thoroughly investigated in human subjects. Overall, our findings highlight the potential of dogs as a suitable model for studying oxidative stress and inflammation markers in clinical investigations related to HC. This is particularly significant given the big difference in incidence rates: 1–2 cases per 1000 dogs annually compared to 1.2–2.4 cases per million humans annually [55].

Hypertriglyceridaemia and hypercholesterolaemia, common biochemical findings in dog with HC [8,56], were confirmed in our study. Excessive GCs may activate both lipolysis and lipogenesis, resulting in an increase in triglycerides hydrolysis in the blood, the generation of free fatty acids in the liver, and the production of VLDL, while simultaneously suppressing FFA oxidation [57,58]. Lipoprotein profile in dogs suffering from HC is not yet fully explored. In Miniature Schnauzer dogs suffering from hypertriglyceridaemia with HC, VLDL and LDL fractions (corresponding to TRL fraction) were unchanged in comparison to healthy subjects, as was HDL [56]. Our study found no differences in TRL fractions, but HDL-1 percentage was higher, with a corresponding decrease in HDL-2,3 in HC dogs. HDL-1 fraction is unique for dogs and this lipoprotein is increased whenever dogs are in hypercholesterolaemia [59]. Due to the absence of cholesterol ester transfer protein activity in dogs, cholesterol esters accumulate in HDL-1, rendering it the most prevalent lipoprotein in dogs. Despite no significant differences in TRL between groups, TRL correlated positively with UA, implying a potential causal relationship. In an observational human study, it was stated that increased serum UA can lead to a higher risk of developing high LDL and hypertriglyceridaemia [60]. One finding suggests that UA triggers the translocation of NADPH oxidase to the mitochondria, resulting in citrate accumulation and the initiation of de novo lipogenesis [61]. TRL was also in correlation with TBARS, which indicates that the increased LP seen in dogs with HC could be related to the dyslipidaemia commonly accompanying this disease. These results are particularly significant given that hyperlipidaemia persisted following trilostane therapy [62]. Dogs with ongoing high levels of lipids are more likely to develop atherosclerosis, even though they are naturally resistant [63]. From a clinical standpoint, more attention must be devoted to the lipid management in dogs, including a special nutrition regime for those suffering from HC.

It is noteworthy that a primary disadvantage of this research is the limited number of animals. Additionally, the study focused exclusively on dogs diagnosed with PHD. To obtain a more comprehensive understanding of canine HC pertaining the scope of our research, future studies should include ADH and iatrogenic forms of HC in larger groups of dogs.

## 5. Conclusions

Our findings indicate that dogs with HC suffer high levels of oxidative damage accompanied by inadequate antioxidant defence. Increased NLR, PLR, SII, and HPT concentrations were identified, although these do not appear to be directly linked to inflammation. In addition, significant alterations in the HDL fraction, along with hypercholesterolaemia and hypertriglyceridaemia were found, which seemingly interlinked oxidative stress and inflammatory indices. Future studies should focus on the effectiveness of antioxidant supplementation in lipid-lowering therapy.

## Figures and Tables

**Table 1:** Demographic and clinical data of the participants.

	Dog with HC (n = 14)	Controls (n = 14)	*p* Value
Age * (years)	10 (7–10)	10.5 (8–13)	0.391
Sex (%)	Male (9): 64.29 Female (5): 35.71	Male (6): 28.57 Female (8): 71.43	0.449
Breeds (n)	Beagle	Beagle	
	Bichon Frise (3)	Maltese dog (2)	
	Coton de Tulear	Golden Retriever (2)	
	French Bulldog (3)	Dachshund (2)	
	Miniature Pinscher (2)	Miniature Schnauzer	
	Mixed breed (2)	Mixed breed (4)	
	Poodle	Poodle	
	Yorkshire Terrier	Pomeranian	
*Clinical data* (%)			
Polydipsia/Polyuria	85.71		
Polyphagia	64.28		
Fatigue	42.85		
Pendulous abdomen	35.71		
Alopecia	35.71		
Thin skin	35.71		

* Data are presented as median (lower quartile–upper quartile).

**Table 2:** Haematology profile of dogs with hypercortisolism (HC) and healthy controls.

Variable	Dog with HC (n = 14)	Controls (n = 14)	Reference Range	*p* Value
WBC (×10^9^/L)	10.47 (7.22–15.7)	8.82 (6.75–11.02)	6–17	0.322
NEUT (×10^9^/L)	9.4 (5.88–13.95)	6.15 (4.49–7.25)	3–12	**0.031**
LYM (×10^9^/L)	0.82 (0.7–0.93)	1.76 (1.65–2.37)	1–4.8	**0.001**
MON (×10^9^/L)	0.39 (0.26–0.78)	0.28 (0.22–0.4)	0.2–1.1	0.212
EOS (×10^9^/L)	0.01 (0.01–0.07)	0.42 (0.2–0.55)	0.1–1.2	**<0.001**
BAS (×10^9^/L)	0.02 (0.01–0.04)	0.01 (0–0.02)	0–0.1	0.274
RBC (×10^12^/L)	6.92 (6.07–7.65)	7.29 (6.91–8.3)	5.5–8.5	0.145
HGB (g/L)	172 (140–179)	179 (161–192)	120–180	0.118
HCT (%)	47.45 (38.95–51)	47.9 (44.8–53.6)	37–55	0.322
PLT (×10^9^/L)	582.5 (425.5–712.5)	270.5 (246–387)	200–500	**0.003**
NLR	9.85 (6.46–28.16)	3.08 (2.28–4.98)	0.74–5.62	**<0.001**
PLR	670.14 (444.47–1511.29)	143.09 (130.7–215.15)	56.41–198.02	**<0.001**
SII	6501(2764–42,392)	391 (252–914)	52.93–1503	**<0.001**

Data are presented as median (lower quartile–upper quartile). Bold numbers indicate significant differences (*p*  <  0.05). WBC: white blood cells; NEUT: neutrophils; LYM: lymphocytes; MON: monocytes; EOS: eosinophils; BAS: basophils; RBC: red blood cells; HGB: haemoglobin; HCT: haematocrit; PLT:platelets; NLR: neutrophil-to-lymphocyte ratio; PLR: platelet-to-lymphocyte ratio; SII: systemic immune–inflammatory index.

**Table 3:** Biochemistry panel of dogs with hypercortisolism (HC) and healthy controls.

Variable	Dog with HC (n = 14)	Controls (n = 14)	Reference Range	*p* Value
Total proteins (g/L)	64.4 (63–73.4)	66.2 (61.6–71)	55–80	0.705
Albumin (g/L)	31.8 (30.2–32.4)	29.7 (29.1–30.2)	25–40	**0.009**
Phosphor (mmol/L)	1.49 (1.4–1.87)	1.3 (1.13–1.36)	0.8–2	**0.005**
Glucose (mmol/L)	6.39 (5.63–8.14)	5.19 (4.36–5.5)	3–6.7	**0.001**
ALT (U/L)	215.1 (149.2–670.3)	50.4 (35.5–73.7)	10–50	**<0.001**
AST (U/L)	44.2 (31.9–74.9)	26.9 (24.1–31.8)	10–58	**0.004**
ALP (U/L)	776.6 (300–2209.2)	41.6 (31.8–56.8)	0–190	**<0.001**
Creatinine (µmol/L)	57.9 (44.4–62.5)	74.2 (64.8–84)	50–169	**<0.001**
Urea (µmol/L)	5.56 (4.48–6.39)	4.99 (4.06–6.27)	3.3–9.2	0.56
CK (U/L)	194.6 (153.7–364.6)	106.9 (91.8–138.5)	40–254	**0.001**
γ-GT (U/L)	31.2 (18.2–144.3)	5.6 (4.1–6.2)	0–7	**<0.001**
BHB (mmol/L)	0.05 (0.03–0.07)	0.04 (0.03–0.04)	-	0.062

Data are presented as median (lower quartile–upper quartile). Bold numbers indicate significance of difference (*p*  <  0.05). ALT: alanine transferase; AST: aspartate transferase; ALP: alkaline phosphatase; CK: creatine kinase; γ–GT: gama–glutamyl transferase; BHB: beta-hydroxybutyrate.

**Table 4:** Oxidative stress and inflammatory markers in dogs with hypercortisolism (HC) and healthy controls.

Variable	Dog with HC (n = 14)	Controls (n = 14)	*p* Value
*Oxidative stress markers*			
TBARS (nmol/mL)	6.52 (5.5–9.31)	4.3 (3.63–6.46)	**0.021**
AOPP (µmol/g)	68.77 (54.29–76.08)	39.64 (29.35–51.72)	**0.001**
TAC (mmol Trolox Equiv/L)	0.14 (0.06–0.46)	0.29 (0.08–0.66)	0.482
GSH (µmol/L)	3.75 (2.57–4.06)	0.96 (0.76–1.81)	**0.001**
PON-1 (U/L)	5.13 (4.72–5.85)	5.11 (4.27–5.4)	0.194
Uric acid (µmol/L)	854 (730–1103)	340 (237–434)	**<0.001**
*Inflammatory markers*			
Haptoglobin (g/L)	1.78 (1.72–1.80)	1.68 (1.65–1.75)	**0.024**
Ceruloplasmin (mg/dL)	6.58 (6.31–7.52)	5.79 (5.64–7.07)	0.291
Homocysteine (µmol/L)	11.88 (9.79–13.31)	14.63 (9.28–18.01)	0.458
Cholinesterase (U/L)	261 (202–317)	217 (206–295)	0.603

Data are presented as median (lower quartile–upper quartile). Bold numbers indicate significance of difference (*p*  <  0.05). TBARS: thiobarbituric acid-reactive substances; AOPP: advanced oxidation protein products; TAC: total antioxidant capacity; GSH: reduced glutathione; PON-1: paraoxonase-1.

**Table 5:** Lipid profile of dogs with hypercortisolism (HC) and healthy controls.

Variable	Dog with HC (n = 14)	Controls (n = 14)	*p* Value
Triglycerides (mmol/L)	2 (1.45–3.97)	0.68 (0.52–0.95)	**<0.001**
Cholesterol (mmol/L)	10.06 (8.96–11.81)	5.18 (4.51–6.3)	**<0.001**
Chylomicrons (%)	5.1 (2.8–7.15)	4.01 (2.28–5.72)	0.511
TRL (%)	8.65 (7.31–12.56)	12.19 (6.92–17.04)	0.285
HDL-1 (%)	44.15 (34.31–51.82)	22.19 (15.59–31.97)	**0.012**
HDL-2,3 (%)	38.44 (32.68–44.77)	60.91 (46.16–70.46)	**0.050**

Data are presented as median (lower quartile–upper quartile). Bold numbers indicate significance of difference (*p*  <  0.05). TRL: triglyceride rich proteins; HDL: high density lipoproteins.

## Data Availability

The data supporting this study’s findings are available from the corresponding author upon reasonable request.
